# Key Knowledge Gaps for *Plasmodium vivax* Control and Elimination

**DOI:** 10.4269/ajtmh.16-0180

**Published:** 2016-12-28

**Authors:** Quique Bassat, Mar Velarde, Ivo Mueller, Jessica Lin, Toby Leslie, Chansuda Wongsrichanalai, J. Kevin Baird

**Affiliations:** 1 ISGlobal, Barcelona Centre for International Health Research (CRESIB), Hospital Clínic–Universitat de Barcelona, Barcelona, Spain.; 2 Centro de Investigação em Saúde de Manhiça (CISM), Maputo, Mozambique.; 3 Population Health and Immunology Division, Walter and Eliza Hall Institute, Victoria, Australia.; 4 Division of Infectious Diseases, University of North Carolina, Chapel Hill, North Carolina.; 5 London School of Hygiene and Tropical Medicine, London, United Kingdom.; 6 Health Protection and Research Organisation, Kabul, Afghanistan.; 7 Independent Scholar, Bangkok, Thailand.; 8 Eijkman-Oxford Clinical Research Unit, Jakarta, Indonesia.; 9 Centre for Tropical Medicine, University of Oxford, Oxford, United Kingdom.

## Abstract

There is inadequate understanding of the biology, pathology, transmission, and control of *Plasmodium vivax*, the geographically most widespread cause of human malaria. During the last decades, study of this species was neglected, in part due to the erroneous belief that it is intrinsically benign. In addition, many technical challenges in culturing the parasite also hampered understanding its fundamental biology and molecular and cellular responses to chemotherapeutics. Research on vivax malaria needs to be substantially expanded over the next decade to accelerate its elimination and eradication. This article summarizes key knowledge gaps identified by researchers, national malaria control programs, and other stakeholders assembled by the World Health Organization to develop strategies for controlling and eliminating vivax malaria. The priorities presented in this article emerged in these technical discussions, and were adopted by expert consensus of the authors. All involved understood the priority placed upon pragmatism in this research agenda, that is, focus upon tools delivering better prevention, diagnosis, treatment, and surveillance of *P. vivax*.

## Introduction

*Plasmodium vivax* is the most geographically widespread malaria parasite species and codominates with *Plasmodium falciparum* as a cause of human malaria. *Plasmodium vivax* infects tens of millions of people each year.^1^ Despite long being regarded as benign, evidence now shows that *P. vivax* is very often associated with severe, life-threatening, and fatal malaria in patients from endemic areas as well as in travelers.^2^

Where control programs have been intensified in coendemic areas, *P. vivax* tends to be more resilient than *P. falciparum*.^3^–^5^ This is due to biological features unique to *P. vivax* enhancing its ability to survive in conditions unsuited to *P. falciparum* and propagate further transmission.^6^ The conventional methods of control historically aimed solely at *P. falciparum* may not suffice. Interventions and strategies aimed specifically at *P. vivax* would greatly accelerate progress against it.^7^

The following specific characteristics set *P. vivax* apart from *P. falciparum* with respect to control and elimination: 1) the hypnozoite leads to relapses, repeated clinical attacks, and onward transmission; 2) gametocytes emerge at an early stage of infection, before onset of illness; 3) transmission by a broad range of anopheline vector species residing in diverse habitats and myriad distinct behaviors relevant to transmitting the parasite; 4) more rapid development in the vector in comparison to *P. falciparum* at the same temperature dampens the population effects of shortening mosquito life span by insecticide-treated net (ITN) or indoor residual spraying (IRS) interventions; and 5) shifts in vector species and behaviors in response to use of long-lasting insecticidal nets (LLINs) and IRS.^8^

To date, the only drug approved to eliminate the hypnozoite of *P. vivax* is primaquine (PQ). This drug sometimes causes life-threatening acute hemolytic anemia in patients with a deficiency in glucose-6-phosphate dehydrogenase (G6PD). G6PD status is essential to safe treatment with PQ, but that diagnosis is rarely available at the health facilities where most malaria patients present. As currently recommended by the World Health Organization (WHO),^9^ PQ requires a 14-day daily treatment course. Counseling strict adherence carries the risk of serious harm in G6PD-deficient patients. These factors, along with fear of PQ by providers and patients alike, sharply erode the utilization and therefore the effectiveness of PQ in practice. PQ is a spectacularly inadequate and ineffective anti-infective drug. Safer and more easily administered drugs are needed to improve treatment of patients infected by *P. vivax* and enable attacking the tenacious and harmful hypnozoite reservoir residing in endemic communities virtually unmolested by any intervention against it.

Scientific progress in *P. vivax* since 1960 has been minimal compared with those in *P. falciparum* because *P. vivax* was inappropriately regarded as a benign infection. Research on *P. falciparum* was the priority because of its high mortality and, in part, because it has been adapted to laboratory cultivation. In contrast, *P. vivax* cannot be maintained in in vitro cultures, hindering fundamental research required to adequately understand its biology, and advance the development of vaccines and treatments.

Research on vivax malaria needs to be substantially expanded over the next decade to address the priorities identified by researchers, national malaria control programs, and other stakeholders. This article aims to summarize the main knowledge gaps that are critical to *P. vivax* control and elimination. In particular, it focuses on the need to develop tools and intervention strategies to achieve more effective prevention, diagnosis, treatment, and surveillance of *P. vivax*.

## Biology, Culture, and Drug Screening

### Develop methods for continuous cultivation of *P. vivax* in vitro.

There is a need to collect, update, share new evidence, and to coordinate research efforts to develop suitable in vitro continuous culture protocols for the *P. vivax* liver stages and for the continuous propagation of its blood stages.

#### Blood-stage culture of *P. vivax*.

In vitro culture of *P. falciparum* was first developed in 1976,^10^ but there are no similar methods for continuous culture of *P. vivax*–infected red blood cells (RBCs).^11^ To date, only short-term cultures of *P. vivax* isolates have been achieved.^12^ Cryopreservation for offsite processing and further studies of culture-adapted *P. vivax* has been shown to be possible,^13^ but still requires standardization and a wider replication. Success in short-term cultivation of *P. vivax* blood stages have already provided key insights into the drug susceptibility and the molecular and cellular biology of this parasite.^11^–^15^ Long-term in vitro culture of *P. vivax* would allow far broader access to laboratory isolates and deeper understanding of the biology of *P. vivax*, and facilitate the development of functional assays. This step is critical in the development of drugs, vaccines, new diagnostic tests, and to obtain gametocytes needed to infect mosquitoes to provide sporozoites for investigation of the liver stages.

#### Liver-stage culture of *P. vivax*.

The main challenge in *P. vivax* control and elimination is the inability to kill the hypnozoite without the significant safety issues of administering 8-aminoquinolines to patients with G6PD deficiency. New and safer hypnozoitocidal therapies would represent an enormous stride forward in eliminating *P. vivax*.

Screening of compounds for activity against hypnozoites currently relies on an animal model (using the closely related parasite, *Plasmodium cynomolgi* in rhesus monkeys). Recent development has allowed drug compounds to be screened using a *P. cynomolgi* liver-stage model.^16^ This in vitro assay represents a major advance and significantly increases the number of molecules that can be tested while substantially reducing costs. In contrast to other such systems, this one is capable of assessing hypnozoitocidal activity rather than just causal prophylactic activity. In other words, it permits assessing impacts upon hypnozoites by allowing their awakening and maturation to active liver schizonts.

Efforts to refine these systems are underway in several laboratories and ongoing challenges include finding stable and receptive hepatocyte cell lines or a reliable source of primary hepatocytes, securing a supply of viable sporozoites (from patients, monkeys, or eventual blood-stage in vitro culture).

A recently developed human liver-chimeric mouse model shows promise both for screening drug candidates and for elucidating hypnozoite biology, but is still relatively low throughput due to expense.^17^

### Investigate key steps in hypnozoite formation, metabolism, and reactivation.

There is no detailed knowledge of the biology of *P. vivax* hypnozoites. It is unknown what determines sporozoite development and differentiation into liver schizonts versus hypnozoites. Several decades ago, Shute and others conducted human challenge trials, the results of which seemed to suggest that the process leading to hypnozoite differentiation was genetically predetermined.^18^ There is also no understanding of whether hypnozoites are metabolically active during their dormancy period, or what triggers their reactivation. Understanding these processes will be essential for developing both novel diagnostic markers that might identify hypnozoite carriers and novel therapies that would impede hypnozoite formation, eliminate the hypnozoite, or trigger the reactivation of hypnozoites to make parasite stages that are susceptible to available antimalarial drugs. The recent development of a humanized mouse system with reactivating hypnozoites^17^ as well as the development of *P. vivax* systems biology resources should aid this endeavor.

### Find important processes in the invasion of reticulocytes and study infected RBC membrane structures.

*Plasmodium vivax* RBC invasion is a complex process which differs from that of *P. falciparum*. After invasion, *P. vivax* actively remodels the RBC membrane creating complex structures such as caveola–vesicle complexes, which are thought to result in the Schuffner's dots visible on Giemsa-stained blood films. The changes result in an extreme flexibility of the membranes of *P. vivax*–infected RBCs. A better understanding of how *P. vivax* invades reticulocytes, why it preferentially does so (as opposed to infecting other more mature RBCs), and how it remodels the RBC membrane after invasion would facilitate the identification of novel vaccine candidate antigens and potential drug targets.

### Reexamine cytoadherent properties of *P. vivax* and their potential role in avoiding spleen clearance.

Much of the morbidity and mortality associated with *P. falciparum* is due to its ability to cytoadhere to endothelial cells, resulting in parasite sequestration in small blood vessels and resultant organ failure. Although *P. vivax* is known to adhere to vascular endothelium, its capacity to do so is relatively limited compared with *P. falciparum*,^19^ and little is known regarding the mechanisms of *P. vivax* cythoadherence to those and other human tissues. There is a need to better understand the role, magnitude, and reversibility of such processes in *P. vivax*, both in (severe) pathology and in the maintenance of chronic *P. vivax* infections. A measure of the parasite sequestered biomass (and location) in vivo is needed, and in particular, *P. vivax* sequestration in organs like bone marrow and the spleen. It has been hypothesized that the bulk of *P. vivax* biomass may occur in those tissues compared with peripheral blood,^20^ and some evidence supports that view.^21^

## Epidemiology

### Develop strategies to distinguish relapses from recrudescence and reinfection.

Current understanding of relapse patterns of different vivax strains is mostly derived from either experimental challenge or clinical studies in soldiers at war in the Pacific carried out over 50 years ago, and these are not likely to be repeated on any similar scale. To better understand the epidemiology of *P. vivax* relapses,^22^ tools or assay strategies that are able to distinguish between recrudescence, new infection, and relapse are needed.^23^ Development of such tools would facilitate assessment of drug efficacy against *P. vivax*, help identify short- and long-latency phenotypes within endemic areas, and pave the way towards identifying hypnozoite diagnostic markers and triggers for relapse. They would also lead to a better understanding of the total disease burden due to relapse, which is likely to have been greatly underestimated. Current strategies for genotyping recurrent vivax infections rely mostly on microsatellite markers, and have revealed that relapses are often multiclonal and usually heterologous (displaying a different genotype compared with the preceding blood-stage infection in the same individual).^24^,^25^ The application of next-generation sequencing technologies has the potential to shed more light on the genetic signatures of relapse (e.g., that they are meiotic siblings)^26^,^27^ that maybe be exploited to develop better genotyping methods.

In parallel, innovative epidemiological studies in well-characterized clinical cohorts suffering relapse are extremely useful to clarify the attributable fraction of relapse to new infections, identify late recurrences, determine the efficacy of antirelapse intervention, and study relapse biology in vivo.^28^ For example, further studies in persons returning to nonendemic areas^29^ followed up for at least 6 months to 1 year, have been conducted in the context of clinical trials of antirelapse therapies.

### Explore the epidemiology of *P. vivax* transmission by characterizing the infective reservoir and the contribution of relapses to sustaining transmission.

Current knowledge of the epidemiology and dynamics of *P. vivax* transmission is limited. *Plasmodium vivax* gametocytes develop earlier in the blood-stage infection cycle and more rapidly than those of *P. falciparum* and often appear before clinical symptoms.^30^ *Plasmodium vivax* gametocytemia tracks asexual parasitemia closely. A small number of studies have shown that *P. vivax* gametocytes are transmitted more efficiently to some anopheline mosquito vectors than in *P. falciparum*, and *P. vivax* gametocytes are transmissible at lower parasite densities.^7^ However, knowledge on the relationship between *P. vivax* gametocyte density and infectivity to the mosquito is weak. For example, in low-transmission settings, the majority of *P. vivax* infections are asymptomatic and of low parasite density, and most of these infections carry gametocytes, but the contribution of this feature to transmission in different epidemiological settings is not well understood. There is a need for further in-depth epidemiological, entomological, and infectivity studies in different epidemiological settings to better understand and target *P. vivax* transmission.

To date, few studies have successfully measured the relative contributions of new mosquito inoculations of *P. vivax* sporozoites versus the activation of dormant hypnozoites to the overall force of blood-stage infection.^31^ Ongoing studies in Papua New Guinea, where *P. vivax* relapses earlier than subtropical strains, indicate that relapses are responsible for up to 80% of *P. vivax* blood infections and contributed substantially to both clinical episodes and transmission.^28^,^32^ Other studies in Thailand and Papua, Indonesia, indicate high prevalence of patent gametocytemia in relapsing infections, highlighting the importance of radical cure for reducing the transmission potential of *P. vivax*.^33^

A better understanding of the contribution of relapses to the infectious reservoir and their importance for sustaining transmission in areas with low transmission and/or long-latency phenotypes is important for the development of rational, evidence-based strategies for combatting and eliminating endemic vivax malaria.

### Investigate the pathophysiology of severe *P. vivax* malaria and its associated global disease burden.

Although long considered benign by malariologists and clinicians, the advent of polymerase chain reaction (PCR) diagnostics in the clinic beginning in the 1990s saw the emergence of a body of evidence associating acute *P. vivax* with severe and fatal outcomes. That evidence has been built into a compelling case for discarding the “benign” label as misleading and dangerous, and instead viewing and managing vivax malaria as a serious and life-threatening infection.^2^

It has been suggested that in settings where malaria is endemic, inadequate therapy and repeated relapses are important contributing factors for severe morbidity and mortality with vivax malaria, particularly that of severe anemia.^21^,^34^ Specific vulnerable groups, such as pregnant women, have also been suggested to be at increased risk of developing severe disease.^35^ Their fetuses are at a 4-fold increase in risk of spontaneous abortion after even a single clinical attack of *P. vivax* in the first trimester.^36^ Many uncertainties still shadow our understanding of the mechanisms through which *P. vivax* may trigger events leading to severe and threatening illness in an as yet unknown proportion of cases.

Further studies assessing the pathophysiology^21^,^37^ in vivo or using postmortem samples^38^ will be required to better understand the mechanisms related to severe disease, and the role of cytoadhesion and other means of sequestration^39^ and/or inflammation in pathogenesis. The role of concomitant infections and comorbidities in the development of severe manifestations still needs to be elucidated.^40^

The WHO's currently proposed working definition for severe *P. vivax* malaria, derived from an expert review meeting on severe malaria essentially states that “the criteria for severe vivax malaria are the same as for adults and children with severe falciparum malaria but with no parasitaemia density thresholds (and without the criterion of hyperparasitaemia).^2^ A more specific expert consensus on standards for case reporting, retrospective and prospective hospital-based studies, and, importantly, village-based assessments of morbidity and mortality by species should be undertaken.^2^,^20^ Efforts should be made to carry out these investigations across representative zones of endemicity.^35^ There is also a need to conduct studies to validate optimal approaches for the management of patients with severe *P. vivax* disease.^40^

### Estimate the burden of *P. vivax* malaria in sub-Saharan Africa.

Duffy-negative phenotype in humans was believed to confer complete protection against *P. vivax* blood-stage parasites, because of the generalized acceptance that parasites could only invade the RBC using the Duffy receptor.^41^,^42^ Nevertheless, there is growing evidence supporting the presence of *P. vivax* infections among Duffy-negative populations in defined areas of sub-Saharan Africa in addition to Madagascar and Mauritania.^43^,^44^ This suggests alternative reticulocyte invasion pathways used by the species^45^ that should be investigated and identified.

In addition, *P. vivax* associated with severe complications has been reported from African countries.^46^–^48^ However, the degree to which different populations living in sub-Saharan Africa are affected by *P. vivax* malaria remains unknown. The consistent finding of confirmed *P. vivax* in Europeans traveling to west and central sub-Saharan Africa^49^ suggests that endemic transmission occurs despite the overwhelming dominance of Duffy negativity among residents. It may be that endemic *P. vivax* indeed occurs but is not prevalent at levels where it may be detected by routine means.

There is a need to estimate whether there is a significant *P. vivax* burden in sub-Saharan African populations to determine whether appropriate screening, management, and surveillance of *P. vivax* cases should be considered as part of program activities, and if so, which diagnostic methods to use for this screening.

### Map prevalence and severity of G6PD deficiency and CYP2D6 poor metabolizers in endemic areas.

The presence of G6PD deficiency is a major operational obstacle to the implementation of antihypnozoite therapy with 8-aminoquinolines. Countries have an increasing understanding of the prevalence and severity of the G6PD genotypes present in their populations, but it is still inadequate to assess risk and guide clinical and public health decision-making. Even less is known about the prevalence of the trait among *P. vivax* patients. As G6PD deficiency is known to be associated with protection against malaria, including *P. vivax*,^50^ its prevalence in *P. vivax* patients is expected to be lower than in the general population, thus altering the risk-benefit ratio of 8-aminoquinoline therapy.^51^ A better understanding of the geographical distribution of severe G6PD-deficiency alleles in general and in *P. vivax* patients in particular would assist development of evidence-based guidelines for 8-aminoquinoline therapy.

Information on G6PD genotypes, biochemical phenotypes, and degree of hemolysis caused by PQ in specific endemic areas will assist in the development of screening and treatment strategies for PQ that mitigate the harm caused by the drug or that of the parasite by withholding it, that is, repeated clinical attacks originating as untreated hypnozoites.

PQ is a prodrug shown to be metabolized by the CYP2D6 isozyme of cytochrome P-450. People with specific CYP2D6 polymorphic alleles fail to metabolize PQ and may thus fail treatment,^52^ leading to false assumptions of PQ tolerance in the parasite. It is currently not clear to what degree CYP2D6 activity impairment may cause therapeutic failure against hypnozoites (or gametocytes of *P. falciparum* as well) or how common such CYP2D6 alleles may be among different *P. vivax*–endemic areas. Better information on the CYP2D6–PQ interaction in different *P. vivax*–endemic regions will help understand the rates of PQ failures and help improve the overall effectiveness of antirelapse therapy in realizing crucial clinical and public health therapeutic objectives.

## Prevention and Transmission Interruption

### Develop integrated vector control interventions that target outdoor and early biting transmission and protect vulnerable populations.

A literature review identified 71 species/species complexes of anopheline mosquitoes with the potential ability to transmit *P. vivax*.^7^ Many of these species are more exophilic and less anthropophilic than highly efficient vectors such as the African vector species, *An. gambiae*. As such, these species are less liable to conventional interventions against the mosquito vector, i.e., IRS and ITN (including LLIN).

Decades of vector control based on indoor insecticidal strategies targeting endophilic mosquitoes have led to shifts to greater outdoor and earlier biting behavior,† †For example, *Anopheles gambiae* (Bioko Island, Kenya, Tanzania), *An. gambiae* s.l. (Nigeria) and *Anopheles funestus* (Tanzania, Benin), *Anopheles farauti* and *Anopheles koliensis* (Papua New Guinea, Solomon Islands), *Anopheles sinensis*, *Anopheles lesteri* (syn. *Anopheles anthropophagus*), and *Anopheles minimus* s.l. (China), *Anopheles dirus*, *Anopheles maculatus* s.l., and *Anopheles minimus* s.l. (Vietnam), *An. dirus* s.l. and *An. minimus* s.l. (Thailand, Vietnam), *Anopheles darlingi* (Brazil, French Guiana, Guatemala), *Anopheles fluviatilis* s.l. (Nepal). or shifts in dominant vector species.‡ ‡For example, from *Anopheles funestus* to *Anopheles gambiae* s.l. (Tanzania-Kenya border, Niger), from *An. gambiae* s.l. to *Anopheles arabiensis* (Niger, Kenya, The Gambia, Tanzania) or *Anopheles merus* (Pemba Island in Tanzania), from *Anopheles koliensis* and *Anopheles punctulatus* to *Anopheles farauti* (Solomon Islands), from *Anopheles minimus* to *Anopheles harrisoni* (Thailand, Vietnam), from *Anopheles lesteri* to *Anopheles sinensis* (China), and from *Anopheles darlingi* to *Anopheles aquasalis* (British Guiana).^53^ In addition, *P. vivax* seems to develop more rapidly in the mosquito than *P. falciparum*, thus limiting the efficacy of methods that aim at shortening the mosquito lifespan, such as ITNs and IRS. As a consequence, methods of control that are broadly more effective in reducing *P. falciparum* transmission, such as ITNs, are less successful in controlling *P. vivax*.^54^

New tools should be adapted to human and vector behaviors. In particular, the development of integrated vector control interventions targeting outdoor and early biting transmission before sleeping time is of high priority. Existing tools need to be optimized (e.g., formulations of long-lasting nonpyrethroid IRS), more appropriate delivery mechanisms have to be devised and tested, and new or innovative control methodologies involving biological and genetic approaches have to be evaluated. These include, among others, safe, novel, longer lasting, and reformulated synthetic insecticides; insecticide-treated durable wall lining, plastic sheeting, and other materials; topical and spatial repellents; insect growth regulators such as pyriproxfen, attractive toxic sugar baits, entomopathogenic fungi and other pathogens, and endosymbionts such as *Wolbachia*.^55^

New tools are needed to prevent *P. vivax* transmission, especially in vulnerable and special at-risk groups such as pregnant and lactating women, infants, or forest workers. In fact, vector control tools may be the only available tools for harm reduction among pregnant women and infants since PQ is contraindicated in these two groups.

### Strengthen entomological research by developing more sensitive vector surveillance tools and standardizing protocols for evaluating vector control interventions.

More sensitive vector surveillance tools—including the use of molecular techniques—are needed to identify which vectors are truly the primary vector species of *P. vivax* so that appropriate and targeted control measures are taken. Well-designed large-scale studies are needed to evaluate the impact of vector control interventions, alone and in combination, in areas where *P. vivax* is dominant. Standardized and universally acceptable protocols that specify the duration of study follow-up, integration of epidemiological and entomological analysis, quality control, and assay systems to determine vector behavior exposed to sublethal insecticide concentrations are needed.

### Develop a vaccine for *P. vivax*.

*Plasmodium vivax* vaccine development has been hampered by inadequate funding, poor laboratory systems for evaluating potential vaccine targets, few vaccine candidates, and very few clinical trials. Each of these obstacles reflects the chronic neglect of *P. vivax* as a research priority since 1960. In response, the November 2013 update to the malaria vaccine technology roadmap has for the first time included a call for new tools to control and eliminate *P. vivax*. The updated malaria vaccine technology roadmap§ §http://www.who.int/immunization/topics/malaria/vaccine_roadmap strategy is to develop vaccines to prevent both clinical disease and its transmission.

Disease-preventing vaccines offer the theoretical advantage that vaccine-induced protective immunity might be boosted by natural exposure to *P. vivax* and could therefore extend the duration of vaccine protection. On the other hand, such vaccines may exacerbate the serious problem of asymptomatic carriers of infection that now seriously impedes elimination in the low-transmission settings typical of *P. vivax* endemicity. However, a transmission-blocking vaccine, if widely applied in conjunction with other control measures, could potentially more readily reduce transmission intensity and more substantially contribute to elimination success. In other words, such a product would prevent both primary blood-stage infections and the establishment of relapse-causing hypnozoites (in other people).

One approach for *P. vivax* disease prevention is to interrupt merozoite invasion of RBCs via the critically important ligand–receptor interaction between the *P. vivax* Duffy-binding protein and the human RBC Duffy antigen. At the time of writing, one of the *P. vivax* vaccine trials in the clinical phase of development is based on this approach. However, the description of cases of *P. vivax* malaria among Duffy antigen–negative individuals indicated that additional parasite ligands (e.g., from the *P. vivax* reticulocyte-binding protein family) might need to be concurrently targeted to achieve an effective antibloodstage vaccine.

In addition, there are currently two ongoing clinical trials based on the *P. vivax* circumsporozoite (CS) protein. Since the CS protein is a malaria antigen involved in sporozoite invasion of hepatocytes and also present on hypnozoites, it is an attractive target against both the sporozoite and intrahepatic parasites.^56^

Vaccine development for preerythrocytic stages of *P. vivax* has been hindered by difficulties conducting sporozoite challenge trials for *P. vivax*. These difficulties include the need to obtain fresh wild-type isolates for each challenge, the possibility of *P. vivax* relapses and the nonavailability of radical cure with complete efficacy to prevent such relapses. Only a few challenge trials have been conducted with limited success.

A Phase 1 safety and immunogenicity trial of a transmission-blocking vaccine based on the sexual stage Pvs25 antigen suffered from undesirable reactogenic side effects. A potentially transformative effort for further development is underway to develop an in vitro assay of serum antibody–based transmission blocking activity by showing that mosquitoes fed on vaccinated individuals do not support the development of *P. vivax* oocysts and sporozoites.

An as yet unfunded and unexplored avenue of vaccination offers potential for success—vaccination by laboratory/factory-reared sporozoites of *P. falciparum* cross-protecting against infection of liver by sporozoites of *P. vivax*. The “live sporozoite vaccine” technology has shown some efficacy with *P. falciparum*,^57^,^58^ and some experiments and data suggest cross-species protection may occur.^59^,^60^

Further development of *P. vivax* vaccines remains a priority as articulated in the updated malaria vaccine technology roadmap. Any vaccine will also have to address the high genetic diversity found in *P. vivax*, which is greater than that found in *P. falciparum*^61^ and maintained even in low-transmission settings. It may be that this area will only move forward if significant new research and development resources are obtained. Without this, the area is likely to remain neglected.

## Case Management

### Improve the evidence base for G6PD deficiency testing and management and develop field-ready point-of-care tests for detecting G6PD deficiency.

Deficiency of G6PD is a genetic trait widely distributed in populations across malaria-endemic regions.^62^ Treatment with 8-aminoquinolines such as PQ can trigger threatening acute hemolytic anemia in patients with G6PD deficiency. Consequently, treatment of *P. vivax* infection with 8-aminoquinolines should be undertaken with knowledge of the individual's G6PD activity status. The use of PQ in pregnant women is contraindicated, not because of the dangers it could cause on G6PD-deficient pregnant women, but because of the uncertainty regarding the G6PD status of the fetus.

At present, the use of a quantitative screening tool that informs the relative degree of enzyme activity is the most direct, accessible, and reliable approach, and remains the gold standard for G6PD-deficiency assessment.^63^ Qualitative tests are also available but only inform whether the person has a certain high level of G6PD in his or her cells. These tests are used as rapid screening tests but usually require a confirmation testing using a quantitative test, with which the actual amount of enzyme activity is measured. Genotyping studies and enzyme activity assays can also be used; however, these methodologies have significant limitations such as false negatives, high costs, or specialized equipment requirements.^63^ In general, however, these methods require fully equipped laboratories and highly trained technical staff and, as such, offer little utility for the vast majority of patients suffering malaria in impoverished and often isolated rural tropical settings.

Data on the performance of G6PD-deficiency-testing products is limited. A systematic evaluation of G6PD-deficiency-testing products that are currently available should be carried out in endemic settings to provide a comparative measure of their performance in a standardized way and to distinguish between adequate and poorly performing tests. Moreover, there is a need to evaluate the effects of acute malaria on G6PD tests, since G6PD-deficient patients who have recently suffered hemolysis may have a normal G6PD test.

To improve G6PD-deficiency testing, further research studies have to be conducted to determine the relationship between G6PD genotype, the level of enzyme activity, and the risk of hemolysis following different dosing regimens of 8-aminoquinolone antimalarials. Current qualitative tests for G6PD deficiency have limitations in guiding treatment with PQ in most heterozygous females.^64^ The phenomenon of lyonization of this X-linked trait explains this problem, that is, a variable proportion of RBCs will express mutant G6PD and fully impaired enzyme activity. The clinical risk this poses to females remains unknown.

Ultimately, there is a need for developing a rapid, inexpensive, and accurate point-of-care test for detecting G6PD deficiency that can be used before PQ treatment in endemic countries.^1^,^63^ This type of test should clearly distinguish the enzyme activity above which it is safe to give therapeutic doses of PQ.^62^,^63^ A point-of- care G6PD quantitative test that functions as a combo test and also measures hemoglobin level simultaneously should become available in 2016, including a built-in thermometer to monitor optimum temperature for the assay (Program for Appropriate Technology in Health-GlaxoSmithKline; http://sites.path.org/dx/malaria/g6pd/). Such a test will require evaluation under field conditions in endemic areas.

### Improve methods for defining antimalarial efficacy.

In areas with chloroquine-resistant *P. vivax*, artemisinin-based combination therapies (ACTs) are recommended by the WHO for the treatment of the acute attack.^9^ This offers a unified ACT-based strategy for treating both *P. falciparum* and *P. vivax* infections in regions where both species are endemic. Unfortunately, current methods for defining antimalarial efficacy in *P. vivax* are more challenging than in *P. falciparum*, due to the confounding effects of relapse episodes (see above). Also, while many molecular markers for drug resistance have been described for *P. falciparum*, well-defined genetic mutations that confer chloroquine resistance in *P. vivax* remain elusive but represent an area of intense study.^65^ Better methods for defining antimalarial efficacy are required to be able to define when chloroquine or ACT efficacy is compromised. Similar methods for measuring antirelapse efficacy of 8-aminoquinolines are also lacking but critical for evaluating new hypnozoitocidal therapies.

### Define the role of ACTs in *P. vivax* treatment.

ACTs have been adopted as the first-line therapy against *P. vivax* in a handful of countries: Cambodia, Indonesia, Vanuatu, Solomon Islands, and Papua New Guinea.^66^

However, more information is required regarding the efficacy and safety of the different ACTs combined with PQ, as well as the activity of PQ against relapse when combined with ACTs instead of chloroquine.^30^,^66^ In addition, cost-effectiveness analyses are required to compare ACTs and chloroquine for vivax malaria and to assess the role of these drugs in vivax malaria control and elimination efforts at different transmission intensities.^66^

### Learn how to administer PQ therapy safely and effectively.

The primary aim of the radical cure of *P. vivax* is to ensure the elimination of both the acute blood-stage infection and the hepatic hypnozoites. This requires administration of two classes of therapies—blood schizontocidal and hypnozoitocidal. Evidence on the efficacy of several widely used PQ regimens is lacking, including those employing ACT as partner drug in radical cure. Antirelapse clinical trials are needed to optimize current policies and practices.^1^ For instance, assessment of short-course, high-dose PQ regimens in studies with long follow-up period is crucial as potential new strategies to improve patient's adherence.^1^ Further studies are needed to define the risk benefit of PQ, since both recurrent episodes of malaria and hemolytic reactions from the drug can result in severe anemia. Evidence is also needed regarding the total dose (which appears to be the key determinant of efficacy) of PQ required to prevent relapses, and this will need to be assessed in different geographical locations.^1^

In addition, data regarding the safety and efficacy of modified PQ regimens (e.g., weekly doses) for individuals with G6PD deficiency are also required.

### Develop guidelines for PQ use in children.

Although PQ pharmacokinetics have been partially characterized in studies of healthy subjects and adult males with malaria, there are few data available in young children, one of the most vulnerable populations at risk of recurrent episodes of vivax malaria.^67^ However, a recent review of the literature reveals 11 clinical studies of PQ therapy that recruited patients under 4 years of age. Although age-stratified tolerability was not reported, the overall safety profile of PQ in G6PD normal individuals was excellent and no worse than for other antimalarial drugs.^67^ Current WHO recommendations, issued in 2015, have recognized this and now allow the use of PQ in children older than 6 months.^68^

Still, more evidence is needed on the efficacy, safety and pharmacokinetics of PQ in young children. The lack of provision of radical cure for infants represents not only a failure to reduce morbidity in this most vulnerable population but also a lost opportunity to have an impact on *P. vivax* transmission.^1^

### Develop alternatives to 8-aminoquinolines.

Tafenoquine, an antimalarial drug invented by the U.S. Army over 30 years ago, is currently the only alternative to PQ being developed with proven efficacy against hypnozoites.^69^ Once licensed, this drug will offer the enormous advantage of a single-dose treatment against relapse. However, as an 8-aminoquinoline, its use remains constrained to the knowledge of the G6PD status of the individual to whom it needs to be administered, similarly to PQ. Innovative G6PD diagnostic technologies may greatly mitigate this problem and permit the full clinical and public health benefits this new therapy promises. Development of effective hypnozoitocidal drugs for preventing *P. vivax* relapses and offering safety in G6PD-deficient patients is a key research priority when considering malaria elimination and eventual eradication. The use of drugs with hypnozoitocidal activity will be crucial not only to prevent relapses in infected patients but also to reduce *P. vivax* transmission potential.

### Develop strategies to prevent relapse in patients unable to receive PQ.

As explained, pregnant or lactating women, their infants, and perhaps those diagnosed with G6PD deficiency cannot receive PQ therapy. CYP2D6-impaired patients may add further to this classification of patients. They require alternatives to PQ as a means of preventing relapse. These may include chemoprophylactic or chemopreventive strategies, for example, intermittent preventive treatment of pregnant women and infants in sub-Saharan Africa. Most of those optimized and validated regimens, however, use sulfadoxine–pyrimethamine, and these are ineffective against *P. vivax*. The research community must conceive, evaluate, optimize, and validate strategies for preventing relapse in patients unable to receive PQ.

## Operational and Health Systems Research

### Improve diagnostics for surveillance and elimination.

A large proportion of *P. vivax* infections are asymptomatic with low and submicroscopic parasite densities.^70^ Studies should assess the public health significance of subpatent *P. vivax* infections,^1^ both in terms of chronic morbidity (e.g., anemia) and their contribution to transmission (see above).

In the context of malaria elimination, new, affordable diagnostics that can rapidly and accurately diagnose low-density *P. vivax* infections are required to detect asymptomatic parasite reservoirs resident in blood. Having a field-ready (i.e., findings rendered within a time frame permitting administration of therapy to those infected) diagnostic tool to detect low levels of parasitemia will facilitate active case detection and help monitor progress towards malaria elimination.^71^ PCR-based techniques have good analytical sensitivity but are difficult to implement under field conditions, whereas loop-mediated isothermal amplification (LAMP) technology represents a more feasible method in resource-limited field settings that still maintains good sensitivity to detect *Plasmodium* parasites.^72^ Further research is needed to assess the cost-effectiveness and implementation challenges of LAMP technology for pursuing active case detection in malaria-elimination settings. A serological diagnosis of acute, subpatent, or latent malaria has not been adequately explored. These technologies have for decades been successfully applied to other infections difficult to diagnose by demonstrating the agent or its DNA/RNA signature.

### Develop strategies to target the hypnozoite reservoir.

It must be recognized that diagnostic tests based on the detection of blood-stage infections do not identify a significant proportion of the reservoir of disease: carriers of the hypnozoite who show no outward signs of disease and who often do not have blood-stage parasitemia. Diagnostic tests that can either directly identify hypnozoite carriers or alternatively identify people at high risk of recurrent parasitemia (e.g., by identifying people with recent exposure to *P. vivax*) would allow better identification and targeting of the infectious reservoir which is key to transmission reduction.

Since there are currently no diagnostic methods that allow the identification of individuals that harbor hypnozoites, mass screening and treatment approaches are likely to be ineffective against *P. vivax* since the hypnozoite reservoir is undetected at the screening and therefore untreated. Mass drug administration (MDA) with an 8-aminoquinoline–containing regimen may thus be a more appropriate approach. However, in low-transmission and preelimination settings, most people may not carry hypnozoites. Given the risk of hemolysis and the difficulty of implementing community-wide G6PD testing, evidence to support alternative MDA strategies is needed, for example, to target high-risk groups (such as those working in endemic areas, travelers, or those with confirmed recent exposure).

### Evaluate the efficacy of current tools against *P. vivax*.

Improving the understanding of the efficacy, effectiveness, cost-effectiveness, and appropriate mix of insecticide-treated nets, preventive treatment, and/or IRS in vivax malaria control is required. These interventions should be examined against endemic vivax malaria in context-specific settings representative of the Americas, south Asia, southeast Asia and the Pacific.

The relatively low transmission and thus large sample sizes required for formal clinical trials makes direct evaluation difficult and costly. Alternative approaches that combine in silico prediction of efficacy using well-validated *P. vivax* mathematical transmission models^73^ with careful evaluation of programmatic implementation studies, may thus be more cost-effective. This will, however, require further development of *P. vivax*–transmission models, in particular of models for low-transmission settings and the role of the hypnozoite reservoir.

### Improve access to antirelapse therapy.

Given the key role of *P. vivax* relapses, it is essential to improve access to antirelapse therapy for all *P. vivax* patients. In many areas, this will not only require implementing evidence-based PQ regimens but also the development approaches for facility-based G6PD testing and/or monitoring for signs of hemolysis.

A high priority will be to conduct operational research to optimize delivery of antirelapse therapy safely and effectively in different settings including strategies to ensure adherence to treatment.

### Develop novel approaches to surveillance and optimize strategies for detection of residual transmission “hot spots.”

As transmission ebbs to very low levels and becomes geographically fragmented, the nature of surveillance systems needs to change. The focus shifts to the detection of foci of residual transmission and different forms of reactive surveillance (triggered by the detection of an index case) should be adopted. At the same time, in those settings, the surveillance strategies have to be used to direct the response.

In low-transmission areas or areas targeting elimination, serological surveys of recent infection may be better suited to the identification of *P. vivax* transmission foci than traditional mass-blood surveys that only detect current patent infections. A different surveillance approach would be to develop genotype maps of *P. vivax* infection at the district level to better understand the local dynamics of *P. vivax* transmission. A formal evaluation of different surveillance approaches and their effectiveness in different epidemiological settings is needed.

### Use modeling to understand current status and future perspectives for vivax malaria.

Epidemiological modeling can be used to assess and quantitatively analyze the current state of the *P. vivax* situation on a global and/or local scale. This will assist in examining the probable impact and improved targeting of interventions previously discussed. This may also assist in optimizing the mix of interventions needed for the interruption of transmission in *P. vivax*–endemic areas.^74^

## Conclusions

Identifying the specific challenges to control and prevention of *P. vivax* is of paramount importance to the global fight against malaria. Without a special focus on vivax malaria,^75^ local elimination will remain elusive and the tremendously ambitious goal of malaria eradication will be more costly, take longer, and its ultimate success less probable. In this respect, intense scientific research is required to adequately address the many knowledge gaps that surround this parasite species.
